# Factors affecting the implementation of a whole school mindfulness program: a qualitative study using the consolidated framework for implementation research

**DOI:** 10.1186/s12913-020-4942-z

**Published:** 2020-02-22

**Authors:** Kristian G. Hudson, Rebecca Lawton, Siobhan Hugh-Jones

**Affiliations:** School of Psychology, University of Leeds, Leeds, England, UK

**Keywords:** Schools, Young people, Mental health, Prevention, Mindfulness, Implementation, implementation frameworks, Consolidated framework for implementation research, CFIR

## Abstract

**Background:**

Preventing the onset of poor mental health in adolescence is an international public health priority. Universal, whole school preventative approaches are valued for their reach, and anti-stigmatising and resilience building principles. Mindfulness approaches to well-being have the potential to be effective when delivered as a whole school approach for both young people and staff. However, despite growing demand, there is little understanding of possible and optimal ways to implement a mindfulness, whole school approach (M-WSA) to well-being. This study aimed to identify the determinants of early implementation success of a M-WSA. We tested the capacity of the Consolidated Framework for Implementation Research (CFIR), to capture the determinants of the implementation of a mental health intervention in a school setting.

**Methods:**

Key members of school staff (*n* = 15) from five UK secondary schools attempting to implement a M-WSA were interviewed at two-time points, 6 months apart, generating a total of 30 interviews. Interviews explored participants’ attitudes, beliefs and experiences around implementing a M-WSA. Interview data were coded as CFIR constructs or other (non CFIR) factors affecting implementation. We also mapped school-reported implementation activity and perceived success over 30 months.

**Results:**

The CFIR captured the implementation activities and challenges well, with 74% of CFIR constructs identifiable in the dataset. Of the 38 CFIR constructs, 11 appeared to distinguish between high and low implementation schools. The most essential construct was school leadership. It strongly distinguished between high and low implementation schools and appeared inter-related with many other distinguishing constructs. Other strongly distinguishing constructs included relative priority, networks and communications, formally appointed implementation leaders, knowledge and beliefs about the intervention, and executing.

**Conclusions:**

Our findings suggest key implementation constructs that schools, commissioners and policy makers should focus on to promote successful early implementation of mental health programs. School leadership is a key construct to target at the outset. The CFIR appears useful for assessing the implementation of mental health programs in UK secondary schools.

## Background

Adolescence is a time of substantial vulnerability to the onset of mental health difficulties. Preventative school-based interventions which reduce risk and enhance protective factors can limit the onset and progression of clinical disorder and promote good mental health [1]. Schools represent an effective platform for the delivery of universal programs available to all pupils.

Mindfulness training (MT) is a promising approach to nurturing good mental health in young people. A number of systematic reviews have shown that short-term, stand-alone (i.e. delivered as a one-off 8 week course) mindfulness based interventions generate medium-sized effects on indicators of psychological health, including anxiety and depressive symptoms, self-esteem, sleep quality, attention and behaviour [2–6]. Being mindful involves managing one’s attention to the present and cultivating awareness of thoughts, feelings and behavioural tendencies, but without over-engaging in them or acting on them unconsciously [7]. Globally, mindfulness is increasingly taught in schools, most often via a group-based program, where mindfulness skills are taught over several weeks either by external trainers or by trained school staff [4]. Whole school approaches (WSA) are the recommended form for many mental health programs promotion (ibid., [8, 9]. A number of reviews have concluded that the multicomponent focus of WSA’s is what makes them more likely than individual classroom-based approaches to result in long-term positive outcomes [8, 10–13]. A WSA utilizes, and seeks to influence, school structures, culture, procedures, ethos and the wider community to secure sustainable improvements and outcomes in young people’s mental health [14].

Despite the evidence for WSA’s recent reviews have shown that whole school interventions adopting a whole school approach are preferable but failing to show impact and suggest this might be due to implementation difficulties [15, 16]. A recent systematic review of mindfulness-based school interventions with early adolescents highlighted the importance of qualitative studies to better inform the implementation of MT [17]. If mindfulness, whole school approach’s (M-WSA’s) are to be effective in schools their implementation will need to be addressed. The impact implementation quality can have on programme outcomes has been reported [18, 19]. A meta-analysis of universal social and emotional skill-based interventions found that interventions with a high quality of implementation produced larger expected outcome effect sizes compared with interventions with a low implementation quality [15]. A more recent meta-analysis of whole school social and emotional learning programs also found that implementation quality was positively associated with program outcome related effect sizes [20].

In a randomised controlled trial (RCT) examining the effectiveness of a mindfulness and yoga intervention in two urban schools, Mendelson et al., [21] found that administrative support, teacher involvement and student engagement were significant predictors of intervention success. Barriers included securing consistent administrative support, teacher engagement, high staff turnover, and overwhelming staff demands. Sibinga et al., [22] tested the efficacy of MT within two Baltimore city public schools, incorporating implementation strategies from previous mental health studies [23]. They found that school leadership buy-in, forming a community partnership and ongoing support for staff were the strategies associated with successful implementation. Dariotis et al., [24] gained the perspectives of both students and teachers involved in a 16-week mindfulness and yoga program around issues of implementation. Program delivery factors, communication with teachers, promoting program buy-in, and program instructor qualities were influential in the successful implementation of mindfulness training.

Implementation research within secondary schools has been less studied. A mindfulness-based universal prevention program for adolescents and for delivery in school (Learning to BREATHE) was evaluated in a fee-paying girls high school [25] and a public co-ed high school [26]. Scheduling challenges, fitting the training into the curriculum and students not being keen to give up their free time to attend the program were noted as barriers to implementation as intended. A more recent study by Meixner et al., [27] explored factors that supported the initial implementation of a mindfulness-based martial arts intervention delivered in a secondary school setting for youth with or at risk for self-regulation challenges. Interviews and focus groups with students, leaders, and teachers found that fidelity to program characteristics, program delivery, and communication were key areas where barriers and facilitators to implementing the program existed. Delivering the program within school hours and having a physical space for the program was perceived to facilitate implementation.

A study by Wilde et al (2019) interviewed senior leadership, school staff and mindfulness trainers at seven secondary schools in the process of implementing MT to understand their experiences of implementation [28]. This study found four main factors which affected the implementation of MT: having champions in the schools driving implementation forward, having resources and time to implement MT, there being a shared understanding of MT and why it is being introduced by school staff, and there being an understanding that implementation occurs through stages and takes time.

Although these findings provide some evidence of possible barriers to MT implementation, further research is needed to know how to actually go about implementing MT using a WSA. Unless implementation information is recorded and reported by MT studies, and clear, definitive implementation outcomes set, it will not be possible to accurately evaluate the effectiveness of school-based MT programs in preventing the onset, or escalation of young people’s mental health difficulties under conditions of the intended, optimal intervention delivery.

The aim of the present study was therefore to identify the determinants of early implementation success of a M-WSA intervention and discover if, how and why does the quality and extent of early implementation of a M-WSA vary across schools? We studied these differences amongst five schools in the UK, one of which was a school for children with profound learning needs. The initial stages of implementation were supported, free of charge, by a national charity, but schools had considerable flexibility in how they responded to that support and what subsequent actions they took. A second aim was to discover how useable and useful an implementation framework known as the Consolidated Framework for Implementation research (CFIR) was in capturing the implementation determinants of a mental health intervention in a school setting. In the last decade over 60 implementation frameworks been identified or developed [29] providing a systematic way to develop, manage and evaluate the implementation of interventions, however, the evidence base for implementation frameworks remains scarce. Very few studies have used implementation frameworks to understand the early barriers and facilitators to implementing school based health programmes.

The CFIR is a comprehensive, organising taxonomy of operationally defined constructs that may impact the implementation success of complex programs. The CFIR defines five domains (intervention characteristics, outer setting, inner setting, characteristics of individuals and process), each with constructs and some sub-constructs which can affect implementation success. To date, the CFIR has been applied to a wide variety of quantitative, qualitative and mixed healthcare related studies pre, post or during implementation for a variety of purposes [30]. We chose the CFIR as it appeared well suited to answering our research question given its’ focus on implementation at multiple levels (individual and organisational) across five domains.

## Methods

### Design

This was a longitudinal qualitative study underpinned by a framework analysis methodology. The work was part of a Doctoral thesis (available at http://etheses.whiterose.ac.uk/id/eprint/22537). Data were collected via face-to-face or telephone interviews at two time points, 6 months apart. These timings were partly determined by the project timeline. Data on school implementation activity (see Context for details), collated by a third party over 3 years, was drawn up to contextualise findings. The study received ethical approval from the University of Leeds Research Ethics Committee (15–0397; 15–0366).

### Context

In 2014, twelve areas in the UK were awarded funding as part of the UK’s National Lottery funded Headstart programme, the aim of which was to build the resilience of young people to mental health difficulties. Cumbria, a region in the north of England, was one of the 12 successful areas and chose to use part of their allocated funding to support schools in the county to implement a M-WSA. The research reported here was conducted by the first author at the invite of the Cumbria region steering group. The Cumbrian offer to schools was to support early implementation of an M-WSA over a 5 year period. The SG’s MT offer provided MT for teachers own wellbeing first, and then interested teachers could go on to train their students. Teachers who accepted the offer received 8 weeks training in mindfulness-based stress reduction [31] after which they are expected to engage in 6 months of mindful self-practice, before receiving an additional 4 days of training on how to deliver a school-based mindfulness training program known as .b for secondary age children, or Paws B for primary age children [32]. After learning .b, they could then start teaching mindfulness to students in their school (https://mindfulnessinschools.org/).

The offer of support from Cumbria was conditional upon schools striving to achieve a shared set of early goals, namely: (i) training teachers in Mindfulness Based Stress Reduction (MBSR), then .b, then delivering mindfulness to either Y7 or Y9 students; (ii) having a way to sustain delivery to new cohorts entering those years; and (iii) ensuring mindfulness had a place in the school curriculum and alongside other core lessons. By way of establishing a relatively crude measure of progress towards these goals, the following information from each interview was ascertained: (a) when a M-WSA was first discussed in school; (b) when MBSR was offered to staff and how many attended; (c) number of staff accessing the .b or paws.b training; and (d) which year group of students and how many had received mindfulness teaching. These data were collated from two sources. One was the interview data. Another was monitoring data undertaken by the Cumbrian project steering group which identified schools’ progress towards a M-WSA over the last three school calendar years. Schools were rated from 1 to 5 (low to high) by the primary researcher. Scores were primarily determined by how many pupils were trained and how far schools were able to sustain this training over the 3 years. The offer was made to schools in September 2014 and monitoring continued until September 2017. Implementation progress was therefore tracked for 3 years. No further stipulations on implementation were made, and schools were free to supplement these with their own implementation plans and activity as much or as little as they wished. Apart from ensuring it was their own staff who trained in mindfulness first, and subsequently delivered mindfulness to their students, schools were not given a plan for how to implement a M-WSA.

The first author interviewed key stakeholders, at two time-points; T1 (February–April 2016) when schools had accepted the Cumbrian offer and had trained their staff (at a point in 2015), and T2, 6 months after the first interview (September – November 2016). Schools were then tracked and their implementation progress recorded. The first author was a male PhD student with substantial experience of conducting qualitative interviews. No relationship was established between the first author and the schools prior to study commencement but the schools were familiar with the SG of which the first author was a member.

### Recruitment

In total, 21 school staff from 5 Cumbrian schools took up the free training for personal well-being (in the form of an 8 week Mindfulness-based Stress Reduction course) being offered by Cumbria [33]. These schools therefore represented a convenience sample for the study. Head teachers at these five schools were contacted asking for permission to advertise the study in their school. All consented, and subsequently, their staff received an email explaining the study and inviting participation (i.e. interview at two time points by a PhD student who wanted to know about their experiences of implementing MT in their schools). Participation was only relevant to those staff who had some engagement with the Cumbrian offer of support (i.e. who had some responsibility for implementation, and /or had opted in to MBSR, and been trained to deliver mindfulness to students).

### Participating schools and staff

Two of the five participating schools were comprehensive schools (state funded and controlled by the local authority), two were academy schools (state funded but free of local authority control), and one was a school for students with special needs. Across these schools, 15 school staff, including 2 head teachers, consented to participate from a total of 23. All participants completed a first and second interview. Table 1 details the key information about participating schools and staff. The percentage of pupils registered for free school meals is included in Table 1 as this is an indicator of deprivation [34].

**Table 1:** Description of participating schools (*n* = 5) and staff (*n* = 15)

School Implementation success (1 being most successful, 5 being the least).	Pupil Demographics	Pupils registered for free school meals (National average: 14.5%)	Year group receiving mindfulness training	Number of Participants who took part in an interview
School 1	1000+ pupils Age: 11–16 Mixed gender	6.8%	Year 7 and 9	5
School 2	1000+ pupils Age: 11–16 Mixed gender	23.5%	Year 7	1
School 3	< 500 pupils Age: 11–18 Mixed gender	5.9%	Year 9	4
School 4	< 1500 pupils Age: 11–18 Mixed gender	5.7%	Year 7	4
School 5	< 150 pupils Age: 3–19 Mixed gender	30.1%	One class	1

### Interview schedule

An interview schedule was designed specifically for this study and aimed to explore participants’ attitudes, beliefs and experiences towards a M-WSA in their school, their reasons for taking part in the teacher MBSR and .b/paws.b training, as well as their views regarding implementation processes and progress of the M-WSA. Indicative interview questions are detailed in Table 2 below. These were not pilot tested.

**Table 2:** Indicative interview questions for the two data collection points

Time point 1	
1. How did the offer of mindfulness training come about?	
2. What was your motivation for taking part in it?	
3. What have you learnt from it, if anything?	
4. What was good / bad about the MBSR course?	
5. Do you practise mindfulness now? Do you use it at work/home?	
6. What do you hope to achieve / will be achieved by bringing mindfulness into school?	
7. Do you have any concerns?	
8. Do you or others have a model in mind for implementation a M-WSA?	
9. What has been happening so far in terms of implementation? Can you outline the steps take / decisions made in this process?	
10. What, do you feel, have been/will be the barriers and facilitators to successful implementation?	
11. What are the next steps?	
12. What have you learned during this process?	
Time point 2	
1. What have been your personal experiences of mindfulness since your training?	
2. How do you feel about it now compared to 6 months ago?	
3. How has the .b training been?	
4. How far has a M-WSA been implemented in your school since we last spoke? Can you outline the steps taken / decisions made so far?	
5. How far have you (or others) achieved what you (or others) set out to do?	
6. What have been the barriers and the facilitators to implementation?	
7. What are the next steps?	
8. What have you learned during this process?	

### Data collection and preparation

In total, 30 interviews were conducted either face-to-face in schools or by telephone. Interviews took place during school hours and staff were pressed for time. Only the participant and researcher were present during interviews. The mean interview time was 21.94 min with a range of 5.51 to 53.02. Interviews were audio recorded using an encrypted voice recorder and transcribed verbatim to playscript standard ready for coding. No field notes were made during or after the interview and transcripts were not returned to participants for comment and/or correction.

### Data Analysis

#### The Consolidated Framework for Implementation Research (CFIR)

**Fig. 1:**
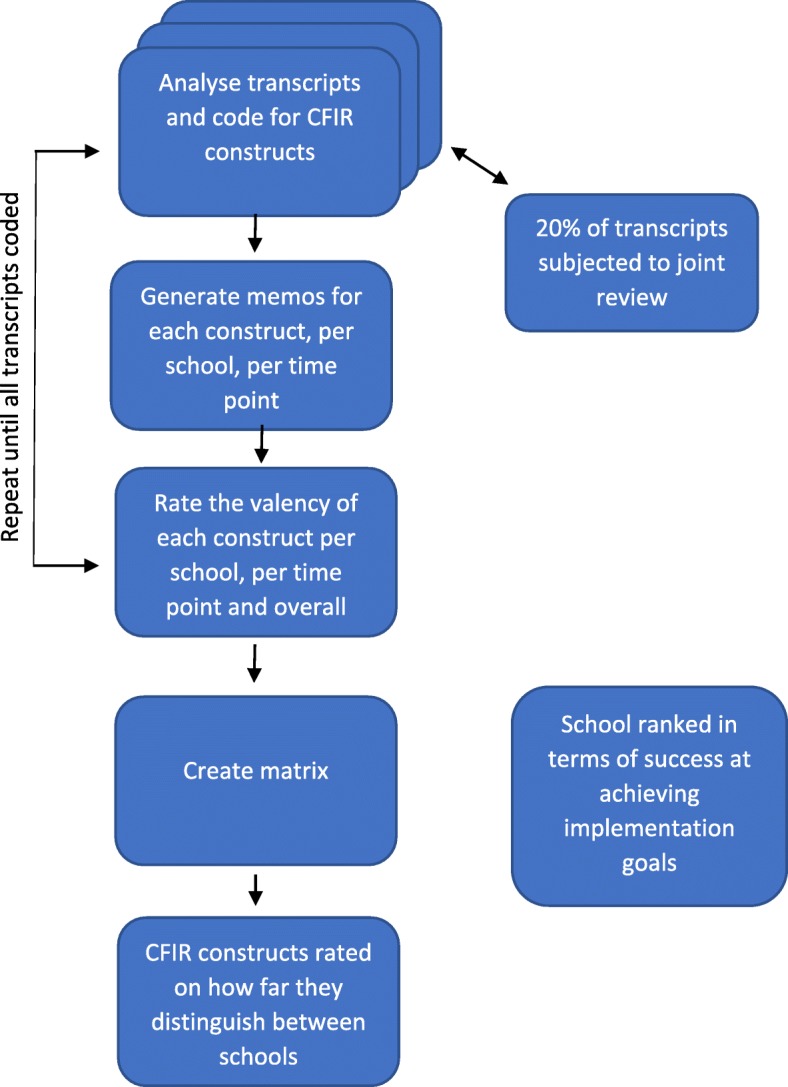
Flow diagram of data analysis

Data analysis was guided by the CFIR. Guidance is available on how to use the framework including definitions of constructs and how to code for them in qualitative data (www.cfirguide.org). We applied the CFIR to our interview data via six analytic stages. The stages included coding for constructs, inter-rater checks, aggregating the data, assigning valence, rating school success in achieving their implementation goals, and matrix creation, all of which are detailed in Fig. 1 and explained in detail in Additional file 1. Inter-rater checks were 98% in agreement and carried out by the first and third authors.

The analysis process meant that every CFIR construct was labelled as strongly distinguishing, weakly distinguishing or not distinguishing between schools.

Valence ratings attempted to capture the extent to which the construct has implicitly or explicitly affected the implementation process. The aggregation of data pertaining to each construct was assigned a valence between − 2 to + 2, representing the direction (positive or negative) and strength (− 2 to + 2) of the construct on implementation. Zero represents no indication of an effect. Mixed effects are rated as X; mixed ratings that were more towards positive or negative impacts were rated, e.g. + 1* / − 1* (See www.cfirguide.org or Additional file 1 for more information).

Ratings for each construct, within each school, ranged from − 2 to + 2. For example, if for a particular construct all five schools were assigned the same score of + 2 this was not deemed to be a distinguishing factor. If, however, for any particular construct, School 1 and School 2 were each assigned + 2, School 3, + 1 and schools 4 and 5, − 2, this was deemed to be a strongly distinguishing factor. Where the pattern was still evident but less pronounced, it would be deemed a weakly distinguishing factor.

This process of analysis supported the possibility of distinguishing between low and high activity schools on the basis of implementation constructs (see Table 3 below). Schools were not invited to provide feedback on the findings but findings were shared with the SG, a mindfulness trainer and a head teacher working at the schools in order to establish validity.

**Table 3:** Ratings assigned to CFIR constructs amongst high and low success schools

	High implementation success			Low implementation success		
School ID	1	2	3	4	5	
1. Intervention characteristics						
Intervention Source	E	E	E	E	E	
Evidence Strength and Quality	+ 2	+ 2	+ 1	+ 2	+ 1	
Relative Advantage	+ 1	Missing	0	+ 1	Missing	
Adaptability	+ 1	+ 2	+ 1	+ 1	+ 2	
Trialability	+ 2	+ 2	+ 2	+ 2	+ 2	
Complexity (reverse rated)	0	0	-1	−2	−2	^a^
Design Quality and Packaging	0	Mixed	Mixed	−1	Mixed	
Cost	0	Missing	0	0	0	
Personal Impact	+ 1	+ 1	+ 1	Mixed	+ 1^a^	
2. Outer setting						
Patient Needs and Resources	+ 1	+ 2	+ 1	+ 1	+ 1	
Cosmopolitanism	Missing	Missing	Missing	Missing	Missing	
Peer Pressure	Missing	Missing	Missing	Missing	Missing	
External Policy and Incentives	+ 1	+ 1	+ 1	+ 1	+ 1	
3. Inner setting						
Structural Characteristics	+ 1	+ 1	X	−2	+ 1	^a^
Networks and Communications	+ 2	+ 2	X	−2	− 2	^b^
Culture	Missing	Missing	Missing	Missing	Missing	
Implementation Climate:						
Tension for change	+ 2	Missing	+ 2	+ 2	Missing	
Compatibility	+ 2	+ 1	+ 1	−1	−1^a^	^a^
Relative priority	+ 2	+ 2	+ 1	−2	−2	^b^
Organizational Incentives and Rewards	Missing	Missing	Missing	Missing	Missing	
Goals and Feedback	0	0	0	0	0	
Learning climate	+ 2	+ 1	Mixed	−1	Mixed	^a^
Readiness for Implementation:						
Leadership Engagement	+ 2	+ 2	+ 2	−2	−2	^b^
Available resources	−1	−2	− 2	−2	− 1	
Access to knowledge and information	Mixed	Mixed	+ 1	Mixed	0	
4. Characterisitics of individuals						
Knowledge and beliefs about the intervention	+ 2	+ 2	+ 2	−2	−1	^b^
Self-efficacy	Missing	Missing	Missing	Missing	Missing	
Individual Stage of Change	Mixed	+ 1	+ 1	Mixed	+ 1	
Individual Identification with Organisation	M	M	M	M	M	
Other Personal Attributes	+ 1^a^	+ 1	Mixed	Mixed	+ 1	
5. Process						
Planning	+ 2	+ 2	+ 1	+ 1	−1	^a^
Engaging:						
Opinion Leaders	Missing	Missing	Missing	Missing	Missing	
Formally Appointed Internal Implementation Leaders	+ 2	+ 2	−1	−2	− 1	^b^
Champions	Missing	Missing	Missing	+ 2	+ 1	
External Change Agents	+ 1	+ 1	+ 1	+ 1	0	
Key Stakeholders	0	0	+ 2	−1	+ 1^a^	
Innovation Participants	+ 1	+ 1	+ 2	+ 2^a^	+ 1	
Executing	+ 2	+ 2	−2	−2	−2	^b^
Reflecting and Evaluating	0	0	0	0	0	

*E* Treated MT as externally developed; *I* Treated MT as internally developed; ‘Mixed’ indicates a mix of positive and negative valency; Missing: indicates no qualitative data was found to correspond to the construct; A ^a^ denoted that the construct weakly distinguished between successful and less successful schools and ^b^ denotes that the construct strongly distinguished between them

## Results

### School success in reaching early implementation goals

All schools were able to identify at the outset which students would be the first to receive the mindfulness training (implementation commitment 1). It was either all pupils in year 7, 9 or in the case of the special school one class of mixed ages received the intervention. All schools at some point were able to deliver the MT to these students and assign curriculum time to this (implementation commitment 3). What differentiated the schools was their ability to ensure MT remained a sustained activity in the school (implementation commitment 2).

### Using the CFIR to code interview data

Coding utilised all 38 CFIR constructs. There was only one aspect of the data for which there was no appropriate CFIR code. This related to when participants spoke about how experiencing mindfulness personally via MBSR and personal practice had led them to become keen advocates of mindfulness. An additional construct ‘personal impact’ was created to capture this.

### Distinguishing CFIR constructs

Table 3 details scoring by school for each of the five CFIR construct domains (Intervention characteristics, Outer setting, Inner setting, Characteristics of individuals, Process) and their sub-categories. Some constructs were found to be more dominant than others in distinguishing between high and low success schools. Schools are presented left to right in decreasing order of success in reaching early implementation goals. A * denoted that the construct weakly distinguished between successful and less successful schools and ** denotes that the construct strongly distinguished between them.

The strongest distinguishing constructs were: *Leadership*, *Relative priority*, *Networks and communications*, *Formally appointed implementation leaders*, *Knowledge and beliefs about the intervention* and *Executing*. Five other constructs exhibited a weak distinguishing effect: S*tructural characteristics, Complexity, Compatibility, Learning climate,* and *Planning*. The remaining 27 constructs did not appear to distinguish between high and low success schools. The following section briefly outlines the strongly distinguishing constructs. Information on the weakly distinguishing constructs is available as an Additional file 3: Table S1.

### Strongly distinguishing constructs

The six strongly distinguishing constructs and supporting extracts are presented below. Many of these constructs were interrelated and *Leadership engagement* in particular seemed to pervade talk around distinguishing constructs.

#### 1 Leadership Engagement

Leadership was a strongly distinguishing construct, ranging from + 2 in Schools 1–3 to − 2 in Schools 4 and 5. In School 1 participants perceived engaged leadership to be fundamental to implementation success, largely due to their decision-making powers: *“because it does take a commitment from her [head teacher] because she is the only person who can make it happen timetable-wise”* (S1, P1,T1: assistant head). The head teacher stated that *“leadership’s always your determining factor”* (S1, P4, T1: head teacher) as “*heads can make things work, or they can block things”*. School 2 was also perceived to have *leadership engagement* as they had appointed an implementation leader with decision-making powers to implement a M-WSA as she saw fit: she explained that to progress implementation *“You do need autonomy”* (P1: T1: head of the SEN). The head teacher in School 3 also showed engagement through the articulation of direction and openness: *“our journey really is about a mindful school and mindful approach to leading a school” (P2: T1: head teacher).*

Although there was early *leadership engagement* in School 4, with senior staff very positive and motivated, they soon disengaged when MBSR was scheduled for a weekend. In addition, the assistant head fundamentally disagreed that teachers needed to practice mindfulness themselves before they could teach it. The training was cancelled, there was no further engagement (or problem-solving) from the head teacher, and no further progress with implementation activity.

In School 5, there was no reported *leadership engagement*, and only one teacher was assigned responsibility for implementation of M-WSA; At T2 the participant commented that it was *“still just myself”* (P1: T2: teacher) and that, in terms of school leadership, there was *“no real support, no real understanding or what the benefits might be”* (P1: T2: teacher).

### Relative Priority

A second strongly distinguishing construct was ‘relative priority’, ranging from + 2 to − 2 across the schools. The level of perceived prioritisation of the intervention appeared strongly associated with schools’ implementation activity. A senior staff member in School 1 conveyed the commitment of the school: *“It’s about that whole system approach, and it’s about driving it forward and making everybody realise that this is definitely part of us, so it’s here to stay, it’s not something that’s just going to be a flash in the pan” (referring to mindfulness and mental health promotion)* (P1: T1: Deputy head). School 1 was also able to maintain the intervention as a high priority over time, despite challenges (e.g. funding cuts).

The implementation lead from School 2 explained the importance of keeping it *“high profile”* (P1: T1: head of SEN) else the intervention could become replaced by new incentives *“something else will come along, and there will be some funding to support that, and that’s what they’ll go for, you know”* (P1: T1: head of SEN). In School 3, there was an awareness of the need to prioritise the intervention so that staff do not *“do lots of training that you never use again”* (P3: T1: Assistant Teacher) but other demands emerged that demoted the priority of implementing a M-WSA:

*“We were hoping to teach Year 7s towards the end of term last year, and that didn’t happen, we ended up being… you’re just overrun with things, and so it was too busy” (P2: T2: Teaching assistant).*

School 4 were under special measures and had improvement targets. They explained that academic attainment was the priority: *“so we’ve got quite a bit of pressure on us to make sure that the kids achieve exam results as well so it’s getting the balance”* (P3: T1). There was a sense of implementation being top-down, where leadership acted and made implementation decisions alone according to what it assumed to be important, whereas, in the more successful schools, the drive to implement was driven more by a set of collective values and involved people from all levels of the organisation. School 4 was also introducing multiple interventions simultaneously. By T2, mindfulness was no longer in the curriculum, “*it just hasn’t happened in the end, so it obviously hasn’t been a priority”* (P1: T1: head of year 7).

In School 5, one participant felt solely responsible for making the intervention a priority, but this was in competition with a major new school curriculum initiative. By T2, the participant had been unable to prioritise MT, *“unfortunately life takes over, as it does at a school and unfortunately I couldn’t prioritise it any more”* (S5, P1: T2: Teacher).

### Networks & Communications

‘Networks and Communications’ was another strongly distinguishing construct. More successful schools had more effective networks of communications “*our team regularly meets on a weekly basis”* (S1: T1, head teacher). “*as a team […] we use each other’s strengths, and we talk, and we work hard”* (S1, P1: T1: Asst head). School 2 participants reported effective communication: *“We’ve had a lot of meetings and discussions about how to go forward” (S2, P1, T1, head of SEN).*

In the less successful schools 4 and 5, more barriers to communication were reported, which was perceived to hinder implementation. In these schools, participants responsible for implementing the intervention had only convoluted communication pathways to senior leaders: *“it goes through me and x to x who then puts it to the senior leadership at their meetings and they then have to decide what is going to happen* (S4: P2: T1: head of year 8:)

Participants felt they could not champion the intervention which was felt to be disempowering and inhibiting of progress. Although Schools 4 and 5 had weekly staff meetings, the intervention was either not bought forward as meriting discussion, or people involved in its implementation were not able to attend these meetings. Schools 1–3 were able to utilise existing effective communication structures to foster implementation.

### Formally Appointed Internal Implementation Leaders

This strongly distinguishing construct linked to *leadership engagement* as leadership was perceived to be influential in the selection of appropriate people to implement the intervention. School 1 reported that the selection of people *“in the best place to have the biggest impact”* (P4: T2: head teacher: 7–8) was a natural response to achieving their intervention specific and global school aims and it was important that these people had some decision making power.

School 2 similarly reported attention to the right set of people

*“Who do you really want to target to go on your courses, to deliver this and take this back? Because that is the key to whether it’s in there long term or not” (P1: T1: Curriculum leader)*

In School 3, the process of selecting staff was perceived to be less evident, and staff exchanged responsibilities for implementation between themselves. Leadership was not involved in this decision. At T2, there was no intervention activity although there had been in the school term previously. In School 4, there was no management decision to appoint implementation leaders. Although two teachers were motivated in School 4, most were not *“because the training was over the weekend” (P2: T2: head of year 8)*. There was a feeling by one participant that: *“perhaps they’re not necessarily the right people to be delivering it anyway”* (P2: T2: head of year 8). Ultimately no one with any decision making power *and* who had a personal role or interest in MT was involved in MT implementation.

In School 5, the lack of communication between management and staff meant that, although management would “*decide which teachers would carry mindfulness out […] these teachers just didn’t want to do it, because they weren’t involved in the decision-making process.”*

### Knowledge & Beliefs about the Innovation

The data painted a picture of staff across the schools holding varied levels of knowledge and beliefs about the intervention and its effectiveness.

However, it was not the nature of knowledge or beliefs that appeared to shape implementation activity but rather who held those beliefs. In the more successful schools, leadership and management reported good understanding of mindfulness and ‘believed in it’: *“There is nothing that would prevent me from doing it, you know, or trying it”* (S2: P1: T1; *head of SEN*). In less successful schools, leadership knowledge and beliefs appeared less favourable to its implementation. In School 4 the assistant head teacher perceived the personal training and practice of mindfulness as an unnecessary condition for teaching it to students, *“I refute the fact that a teacher who doesn’t find it useful as a person can’t actually put over to children that they might find it useful because of course we can do that* (S4, P4: T1). The leadership team in School 5 was perceived by participants to have a poor understanding of mindfulness and little belief in its potential for their students.

Thus, when individuals with power in the school did not believe the intervention would help the students or did not value the training process implementation was weaker.

### Executing

Executing was the final strongly distinguishing factor and refers to carrying out or accomplishing the implementation according to plan*.* Participants in more successful schools tended to perceive that their plans had been executed more effectively than participants in lower activity schools. And this construct corroborated well with the SG monitoring data.

Five other constructs exhibited a weak distinguishing effect: S*tructural characteristics, Complexity, Compatibility, Learning climate,* and *Planning* and these are presented and explained in Additional file 1.

## Discussion

The aim of this study was to identify if, how and why the quality and extent of early implementation of a M-WSA varies across schools as well as how usable and useful the CFIR might be in capturing the determinants of implementation success of a mental health intervention in a school setting. The CFIR demonstrated a high degree of applicability to the data with 11 constructs distinguishing between high success and low success schools over time. Six of these constructs were strongly distinguishing. In less successful schools, distinguishing constructs tended to fluctuate towards a more negative valency over time.

Leadership was arguably the most influential construct because it was consistently reported as determining the valence (whether positively or negatively) of the other constructs. Wilde et al [28] also found that support from leadership towards those responsible for implementing MT in their school was perceived to contribute to implementation success. A lack of leadership engagement can substantially reduce the chance of successful school program implementation [35–38]. School leaders have been shown to be key facilitators of implementing mental health programs [37] and comprehensive school reform efforts (similar to WSA’s) elsewhere [39]. Successful schools in the present study had more engaged leaders who tended to support and encourage staff in the use of the intervention more than less engaged leaders. In less successful schools, there was little encouragement from school leaders during the initial period of ‘buy in’ which can hinder the use of research based knowledge in secondary schools [36]. Leaders in more successful schools believed in their staffs’ abilities, communicated clear goals regarding implementation, choose MT because they felt it was compatible with their school’s needs and in the face of funding cuts ensured resources were available to continue implementing MT. These have all been reported by Wong and Rutledge [40] as elements of ‘strong’ rather than ‘weak’ leadership. It seems that strong leaders are the ones who create a school climate conducive to change [41].

Leaders in schools 1 and 2 actively employed staff to oversee the implementation of mindfulness (as captured by the CFIR construct ‘formally appointed implementation leaders’). However leaders in these schools were careful to make sure these staff had autonomy and decision making power. By selecting staff with decision-making power school leaders created a culture of ‘shared leadership’. In schools 3, 4 and 5, no formalised selection of staff occurred and it was left up to staff to volunteer themselves. The staff that did volunteer themselves to train in mindfulness and implement it had no decision-making power. *This is a slightly different finding to Wilde* et al.*,* [28] *who found that schools needed a committed individual, supported by leadership, to champion MT in order to drive it forward but the authors did not find evidence that these individuals needed autonomy or decision-making power themselves. The important role of formally appointed implementation leaders and their need for autonomy in decision-making power became much clearer by stage II and had Wilde* et al.*,* [28] *interviewed school staff again at a later date perhaps their need for personal autonomy may have become clearer. Wilde* et al.*,* [28] did report that when implementation leads had no obstructions from staff higher up the hierarchy; implementation was more readily achieved. Allowing for some level of distributed leadership (where leadership practices are distributed across a number of individuals in a school) is thought to be a key way school leadership can ensure change processes are successful in their schools and sustained school improvement programs can be accomplished at scale [42].

Previous qualitative research into the barriers to implementing a trauma based mental health program across 8 schools in the USA found that ‘competing responsibilities’ was the strongest barrier to implementation [37] and leaders in successful schools were able to protect mindfulness from these. Leadership in schools 1,2 and 3 portrayed a more ‘adaptive’ leadership style than leaders in school 4 and 5 where dialogue, involvement, negotiation and collaboration were used to develop solutions to barriers when no ready-made, routine solution was available [43]. A strong perception of school mission, vision and goals around MT implementation, e.g. “We’re aiming to be a mindful school” was also more evident in high success than low success schools which can make the implementation of EBP’s more likely [44].

*Wilde* et al.*,* [28] *found that the perceptions of school staff towards mindfulness was perceived as being important to implementation. The present study found the CFIR construct ‘knowledge and beliefs’ to be an important implementation construct within the data. However, the knowledge and beliefs of school leaders in regards to MT had a far greater impact on implementation than the knowledge and beliefs of staff. It was the knowledge and beliefs of leadership which made the difference, not necessarily the knowledge and beliefs of the rest of the school staff, which tended to vary considerably within each school but had less impact on implementation and the police of their school leaders.*

Creating a well specified plan is an important first step to any implementation process in schools [45], and all the schools in this study had an initial implementation plan (planning). However, more successful schools were better at maintaining this plan over time. They also tended to execute (executing) MT implementation more effectively than lower success schools.

Leaders in successful schools tended to have positive personal beliefs about the effectiveness and suitability of MT to their school as well as an accurate understanding of it (construct: knowledge and beliefs) whereas leaders in the less successful schools did not. Headteacher beliefs have been shown to impact implementation of school health programs elsewhere [46]. This highlights the need for program designers and external program funders to ensure school leaders are provided with accurate and easy to digest evidence about an intervention and that any myths around it are challenged.

### Using the CFIR for a school mental health intervention

For the most part, applying CFIR constructs was straight-forward, but there were instances where deciding which construct to apply was difficult, e.g. distinguishing between complexity and compatibility, or design quality and packaging as opposed to access to knowledge and information. For researchers using the CFIR, the online technical assistance from www.cfirguide.org can be an invaluable source of guidance. Through discussion, raters were able to agree on which construct to assign.

A key finding from the interview data was the ‘need for momentum maintained over time’ in order to achieve implementation success. The CFIR does not have a construct which captures this well. The CFIR seems usable and useful for analysing a snapshot of implementation or one point in the implementation cycle but is a less useful coding system for examining the degree of sustained implementation. Conducting interviews at two time points allowed us to capture the idea of growth and momentum.

The construct ‘personal experience’ needed to be created to capture the requirement that trainers personally experience the intervention before delivering it. This strongly influenced the valency of ‘knowledge and beliefs’ and ‘evidence, strength and quality’ amongst participants.

Notably, the construct of ‘culture’ was not assigned during the coding process, i.e. the norms, values, and basic assumptions of a given organization [47]. This was surprising given previous findings of the importance of organisational culture to the implementation of school health programs [48]. It is possible that the importance of culture was implicit rather than explicit in interviewee accounts, or that it was more easily coded as other ‘inner setting’ constructs that could be seen as proxies for culture, e.g. learning climate or networks and communications.

### Study Evaluation

This is the first study to apply the CFIR to school-based implementation research. The constructs are considered applicable to public health implementation activities in general [49] and we found its application to school settings a useful and fluid process. Using the CFIR allowed for results to be generalizable and therefore applicable to other school settings using other school mental health programs, something which has been advocated as a key reason to involve implementation theories and frameworks in implementation research [30]. Our study gives an indication of the facilitators and barriers to the early implementation of a M-WSA.

The number of schools in the study was small, thus limiting generalisability. However, according to the concept of information power [50] we felt the interviewee sample was appropriate for qualitative analysis. Although a study may miss something important if its sample size is too small [51] information power indicates that the more information a sample holds, relevant for the actual study, the lower the number of participants needed. We had a narrow research aim and a group of participants with a diversity of experiences which we were able to interview twice over 6 months. We therefore felt a sample size of 15 was sufficient. We did not interview other stakeholders (i.e. students, parents, staff) not involved in implementation. Analysts were not blind to the implementation success of schools, so there is a possibility of bias in the ratings. This study also examined schools in a particular context (i.e. where a charity, Headstart offered schools a range of programs to improve the resilience of 10–16 year olds, and it may be that different offers of support, within different contexts hold different barriers and facilitators to implementation). Although this study included a school for children with profound learning needs, there is not much implementation research which has addressed this population. Further research will be needed to understand how MT might need to be modified to be delivered into these settings. In particular the contexts and needs of different populations will need to be examined as they could influence uptake and sustainability.

Care was taken to ensure interview questions did not tap into specific constructs, otherwise there may have been a risk of bias, whereby interview questions increased the chance of some constructs appearing in the data over others. For example participants were never specifically asked about the importance of leadership engagement or ensuring the program was made a priority. The three conditional implementation goals set out by Headstart which schools had to agree to in order to receive the MT offer may have had an impact on which constructs arose from the data. For example, schools had to agree to train teachers first in MBSR and then .b. This may have, for example, impacted the non-distinguishing construct planning.

## Conclusions

The CFIR seems to be useful for identifying barriers and facilitator to EBP’s in schools. The results from this study inform how we understand outcomes of ‘services’ delivered in schools and point to the need for system organisation in these complex settings to ensure EBPs reach target users. The results suggest that in order to maximise the implementation of mental health programs in schools, it may be worth targeting school leaders. Leaders who want to implement MT need to take responsibility for ensuring the stages of implementation are supported and achieved in school [41]. Future studies could, therefore, seek to understand whether school leaders can be trained to apply findings from implementation science research to the implementation decisions they make when implementing an EBP. Future studies could also explore whether the behaviours of leadership in schools can be steered towards being more in support of successful implementation. Behaviour change is what drives implementation [52]. In order to navigate the implementation process, the National Implementation Research Network (NIRN) recommends school leaders adopt both technical and adaptive leadership styles as different implementation problems often require different leadership approaches [19, 53].

Who should be responsible for implementation is less clear. It is possible that a concerted effort on the part of program designers, program funders and school leadership might be needed to ensure schools have the capacity and knowledge to implement mental health programs well. This idea is echoed by Metz [54] who suggests successful uptake of EBP’s across service settings will require ‘co-creation’.

## Supplementary information

**Additional file 1:** Detailed explanation of Method.

**Additional file 2:** Interview guides used for the two data collection points created specifically for this study.

**Additional file 3: Table S1.** Additional weakly distinguishing constructs found from analysis.

## Data Availability

The datasets used and/or analysed during the current study are available from the corresponding author on reasonable request.

## Abbreviations

CFIR: Consolidated Framework for Implementation Research
EBP: Evidenced based practice
MBSR: Mindfulness Based Stress Reduction
MT: Mindfulness training
M-WSA: Mindfulness, whole school approach
NIRN: National Implementation Research Network
P1–15: Participant number
RCT: Randomised Controlled Trial
S1–5: School number
SEN: Special Educational Needs
SG: Steering Group
T1/T2: Time 1 / Time 2
UK: United Kingdom
USA: United States of America
WSA: Whole School Approach
