# Supplementary material for: Factors affecting the implementation of a whole school mindfulness program: a qualitative study using the consolidated framework for implementation research
Source: BMC Health Serv Res. 2020 Feb 22;20:133. doi: 10.1186/s12913-020-4942-z (PMC7036167; doi:10.1186/s12913-020-4942-z)
Supplement: Supplementary file 1 — Additional file 1: Detailed explanation of Method. [file 12913_2020_4942_MOESM1_ESM.docx]

Supplementary file 1

Detailed explanation of Method:

Stage1: Coding for constructs

Coding at this stage involved attempting to accurately assign CFIR constructs to the data. All 38 constructs, from all five domains were successfully applied. T1 and T2 transcripts for each participant were coded separately by the first author. Proceeding line-by-line, implementation relevant sections of talk were identified. Where possible, these were assigned a CFIR construct code if the data met the inclusion criteria as specified in the CFIR codebook. In cases where it was not possible, a new code was generated.

Stage 2: Inter-rater checks

The third author reviewed, with the first author, the first coding across a random 20% of the transcripts. All assigned constructs were checked for appropriate fit. In total, 33 constructs across 194 instances of implementation-relevant talk were reviewed. Only 4 instances of disagreement were identified (Inter-rater checks were therefor 98% in agreement). Consensus was reached through discussion. We established validity by feeding back the findings to the steering group and consulting with a mindfulness trainer and a head teacher based at one of the schools.

Stage 3: Aggregating the data

We used a case memo template (http://www.cfirguide.org), and memo construction proceeded as follows: (i) combining the coding of T1 data for each school’s participants; (ii) combining the coding of T2 data for each school’s participants; (iii) based on these, producing one memo for each school across both time points. Memos were organised by CFIR constructs, with each construct supported by summary statements and interview extracts. This resulted in 5 memos in total, one for each of the 5 participating schools, which included large amounts of data

Stage 4: Assigning valence

During the data aggregation stage, judgments were made as to the impact of each construct on implementation in each school at T1 and T2 separately, and also at both times combined. Cfirguide.org provides criteria for judging the valence of assigned CFIR constructs. Valence ratings attempt to capture the extent to which the construct has implicitly or explicitly affected the implementation process. Once collated and summarised, the aggregation of data pertaining to each construct can be assigned a valence between -2 to +2, representing the direction (positive or negative) and strength (-2 to +2) of the construct on implementation. Zero represents no indication of an effect. Mixed effects are rated as X; mixed ratings that were more towards positive or negative impacts are rated e.g. +1* / -1*.

Using these ratings, we produced an overall valence score per construct, per school, both for T1, T2 and overall, meaning we could capture the effect of each construct on implementation over time and more easily produce an overall valence of each construct per school.

## Stage 5: Rating school success in achieving their implementation goals

This stage involved examining how far each school had achieved their early implementation goals. The offer of support from Cumbria was conditional upon schools striving to achieving a shared set of early goals, namely: (i) training teachers in MBSR, then .b, then delivering mindfulness to either Y7 or Y9 students; (ii) having a way to sustain delivery to new cohorts entering those years; and (iii) ensuring mindfulness had a place in the school curriculum and alongside other core lessons. By way of establishing a relatively crude measure of progress towards these goals, we ascertained the following information: (a) when a M-WSA was first discussed in school; (b) when MBSR was offered to staff and how many attended; (c) number of staff accessing the .b or paws.b training; and (d) which year group of students had received mindfulness teaching and how many. These data were collated from two sources. One was our interview data. Another was monitoring data undertaken by the Cumbrian project steering group which identified schools’ progress towards a M-WSA over the last three school calendar years. The offer was made to schools in September 2014 and monitoring continued until September 2017. Schools were rated from 1-5 (low to high) by the primary researcher. Scores were primarily determined by how many pupils were trained and how far schools were able to sustain this training over the three years.

## Stage 6: Matrix creation

In this final stage, a matrix template was created that listed the ratings for each CFIR construct per school, combined across time points. This allowed us to identify whether certain constructs appeared more dominant (i.e. had the strongest valency and were reported most frequently), and whether these constructs distinguished between successful and less successful schools in terms of reaching early implementation goals. Constructs were therefore labelled as strongly distinguishing, weakly distinguishing or not distinguishing at all in this regard. For example, if for a particular construct all five schools were assigned the same score of +2 this was not deemed to be a distinguishing factor. If, however, for any particular construct, School 1 and School 2 were each assigned +2, School 3, a +1 and schools 4 and 5, a -2, this was deemed to be a strongly distinguishing factor. Where the pattern was still evident but less pronounced, it would be deemed a weakly distinguishing factor.
