# Supplementary material for: Factors affecting the implementation of a whole school mindfulness program: a qualitative study using the consolidated framework for implementation research
Source: BMC Health Serv Res. 2020 Feb 22;20:133. doi: 10.1186/s12913-020-4942-z (PMC7036167; doi:10.1186/s12913-020-4942-z)
Supplement: Supplementary file 2 — Additional file 2: Interview guides used for the two data collection points created specifically for this study. [file 12913_2020_4942_MOESM2_ESM.docx]

**Supplementary file 2**

**Interview guides used for the two data collection points created specifically for this study.**

**Time point 1**

1. How did the offer of mindfulness training come about?
2. What was your motivation for taking part in it?
3. What have you learnt from it, if anything?
4. What was good / bad about the MBSR course?
5. Do you practise mindfulness now? Do you use it at work/home?
6. What do you hope to achieve / will be achieved by bringing mindfulness into school?
7. Do you have any concerns?
8. Do you or others have a model in mind for implementation a M-WSA?
9. What has been happening so far in terms of implementation? Can you outline the steps take / decisions made in this process?
10. What, do you feel, have been/will be the barriers and facilitators to successful implementation?
11. What are the next steps?
12. What have you learned during this process?

**Time point 2**

1. What have been your personal experiences of mindfulness since your training?
2. How do you feel about it now compared to 6 months ago?
3. How has the .b training been?
4. How far has a M-WSA been implemented in your school since we last spoke? Can you outline the steps taken / decisions made so far?
5. How far have you (or others) achieved what you (or others) set out to do?
6. What have been the barriers and the facilitators to implementation?
7. What are the next steps?
8. What have you learned during this process?
