# Supplementary material for: Factors affecting the implementation of a whole school mindfulness program: a qualitative study using the consolidated framework for implementation research
Source: BMC Health Serv Res. 2020 Feb 22;20:133. doi: 10.1186/s12913-020-4942-z (PMC7036167; doi:10.1186/s12913-020-4942-z)
Supplement: Supplementary file 3 — Additional file 3: Table S1. Additional weakly distinguishing constructs found from analysis. [file 12913_2020_4942_MOESM3_ESM.doc]

**Table S1:** Additional weakly distinguishing constructs found from analysis

| School | Weakly Distinguishing Constructs | | | | |
| --- | --- | --- | --- | --- | --- |
|  | 7.**Structural characteristics**  *The social architecture, age, maturity, and size of an organization* | 8. **Learning Climate**  *A climate in which: a) leaders express their own fallibility and need for team members’ assistance; b) team members feel they are essential, valued and knowledgeable in the change process; c)individuals feel psychologically safe to try new methods d) there is time to reflect and evaluate* | 9. **Complexity**  *Perceived intricacy or difficulty of *the innovation*, reflected by duration, scope, radicalness, disruptiveness, centrality, and intricacy and number of steps required to implement* | 10. **Compatibility**  *The degree of tangible fit between meaning and values attached to the intervention by involved individuals, how those align with individuals’ own norms, values, and perceived risks and needs, and how the intervention fits with existing workflows and systems* | 11. **Planning**  *The degree to which a scheme or method of behaviour and tasks for implementing an intervention are developed in advance and the quality of those schemes or methods* |
| School 1 | Continuity in structure (i.e. no restructures). Head teacher supported by deputy head teachers and implementation team. | All participants given clear roles in terms of implementing mindfulness. Participants were valued by leadership *“I couldn’t do it without (name of team member)”* (P1: T1: Asst Head: 417-418), able to trial mindfulness, make changes as appropriate as well as put forward new ideas *“some of our job is to find different things that we can bring in*” (P1: T1: Asst Head: 555-556) | Participants perceived the training as highly complex i.e. in its duration, scope, training expectations. Due to good teamwork, making MT a priority, good organisation, planning and execution the complexity of mindfulness did not hinder its implementation. | MT perceived as compatible with their current timetabling plan *“I have been able to just get on and put it in, and we’ve had the support to make sure that we’ve got timetable time,” (P1: T1: Assistant head: 171-172).* | Participants described collective, mindfulness focused planning meetings, *“we were having a mindfulness meeting about where we’re going next with it”* (School 1: P1: stage II: assistant head:). Decisions were made together: *“we put together a little plan”* (School 1: P1: stage I: assistant head:) and participants knew which direction the school was headed in, “*I know next year we’re starting with Year 7”.* |
| School 2 | Underwent restructuring. Implementation leader ensured *“resilience in the system”* *(P1: T2: head of SN, 15-17)* by training two more teachers. | Teachers felt valued, were appreciated by leadership, able to make appropriate changes to MT when needed *“I’ve brought the bigger picture in and we’ve had to put some fillers in”* (P1: T1: head of SN: 58-60), as well as take time to reflect *“it’s been carefully thought through”* (P1: T1: head of SN: 483-484) | The intervention was experienced as unusual so required a bit more thought and planning to teach the students but there was no evidence that the complexity of MT diminished its implementation | Successfully introduced MT to year 7’s but were unable to introduce it to year nines as originally planned due to academic commitments, *“you can imagine in a secondary school once you get to Year 9 and you’re onto your GCSE years that curriculum time is absolutely precious. So we weren’t able to take that into the Year 9 and above which is where that .b was aimed at really (P1: T2: Head of SN: Teacher: 78-84)”.* | There was a perception of a collective effort of where to take MT next, *“Today was the S band. The next round is going to be with the W band and they’re the high ability students”* (P1: T1: head of the SN: 374-376). |
| School 3 | Relatively stable environment, no restructures but centralised decision-making and one head teacher acting alone slowed down the implementation of MT. | Teachers felt supported *“we’re not doing it in isolation so there’s quite a few of us supporting each other”* (School 3: P3: T1: Teaching assistant 252-253) but staff felt they couldn’t make appropriate changes to MT and that there wasn’t time to reflect, *“we need time for staff to offload and there isn’t that at the moment and able to make changes to lessons”.* By T2 interviews, evidence of a learning climate had decreased as teachers were unaware of plan going forward, *“I trust that Andy knows what he's doing in terms of timetabling it in, and I'd be happy to, you know, to carry on with the teaching of it”.* | Training for the teachers was perceived to be a long and complex process with some staff struggling to complete the self-practice component. Leadership made a big effort to ensure staff could attend the training, *“So we have to publicise how we would manage their directive time commitment to enable them to go to their mindfulness sessions which is 16 hours, and offset that commitment with their other directed time hours (P2: T1: Head teacher, 67-69).* | Perceived MT to be compatible with their workflows *“I mean Andy will put it in the timetable and it’s during my PSHE lessons so it’s great, it’s just fits in really well there” (P3: T2: teacher: 66-67)* but they did not manage to maintain its presence in the timetable after its initial introduction in calendar year one. Putting MT in the timetable was believed to cause some tension amongst teachers not involved in MT, “he was really angry about that” (P4: T2: teaching assistant: 179-182). | There was a collective sense of meetings being held and decisions being made in regards to who to teach first: *“we have a team come together and we put together a plan for the spring term” (P2: TI: head teacher: 41-43).* By T2 interviews perception was that collective planning and awareness of future steps had stopped, “May be the head has decided not to continue things, he felt he couldn’t justify, but I don’t know I’ve not had that conversation with him. So this year nobody has had mindfulness” (P4: T2: Teacher: 40-42). |
| School 4 | Underwent restructuring, implementation roles disappeared. Re-structure led to a shift away from mental health to academic achievement: *“there is no emotional type focus or anything like that left in the school” (P2: T2: Head of year 7).* | Clear evidence of teachers feeling unsupported, *“I don’t know what the solution is, I need some advice really”* (School 4: P2: T1: head of year 8: 59-60). There was little time to reflect and evaluate, “(we are) *kind of doing everything off the cuff, (*rather than) *planning properly in advance”* (School 4: P3: T1: Inclusion Leader, 71-73). | Staff found the extent of the training difficult to abide by and many did not complete the self-practice requirements. MT was perceived as a “*massive commitment”,* *(School 4: P3: T1: Inclusion Leader, 548)* and that *“having it like four till six is a big cut in to people’s times” (543).* Large numbers of teachers dropped off the course. | There was evidence of an incompatibility of MT with some participant’s values including leadership: *“I refute the fact that a teacher who doesn’t find it (MT) useful as a person can’t actually put over to children that they might find it useful, because of course we can do that”* (P4: T1: deputy head: 36-38). | Although participants described the school as having successfully piloted MT with a “*Year 7 personal development class”* (P3: TI: execution leader: 33), they were not able to refine it later on and the next year 7’s did not receive MT. |
| School 5 | Stable structure; no effect on implementation. | Reluctance from leadership to include staff who delivered MT in management meetings, “*There was a suggestion to make people that have done the MT to put them on the management team* *and Headstart would pay for that…the head wasn’t keen on that idea at all”* *(P1: T2: Teacher: 35-37).* | In school 5, the complexity of the MT was largely too much for its special needs students, *“They don't get it, I must admit, the youngsters with learning difficulties aren't getting all of the information, but they do get the breathing part of it and the relaxing part of it, and I, yeah, and I use it myself pretty much a daily, a daily practice”* (School 5: P1: T1: Teacher: 62-65). | MT was compatible with workflows but not with the students: *“it just didn’t fit. It just didn’t fit our youngsters, it was never designed for youngsters with learning difficulties”* (School 5: P1: T2: teacher: 3-4). They did not keep it in the curriculum after year one. | Participant indicated that there was very little collective sense of a plan going forward. S/he had taught her own students: *“I use it with myself and I have diluted a version in school for the youngsters I work with” (P1: T1: Teacher: 62-63). S*he described a plan of how to proceed, but there was no indication or effort from leadership to acknowledge this plan and put it into action. |
| Summary | Low success schools were affected by change, had no succession planning or risk management. | High success schools reported demonstrating and fostering more aspects of a learning climate than low success schools. | All schools felt MT was a highly complex intervention. Its duration, scope, training expectations (six months of self-practice, eight weeks training), made implementation difficult. High success schools were able to deal with this complexity more than low success schools. | More successful schools perceived MT to be more compatible with their workflows and/or values. | Higher success schools had a greater collective sense of the initial plan and future plans moving forward than lower success schools |
